# Buffalo Hospital of the Sisters of Charity

**Published:** 1852-09

**Authors:** 


*Buffalo Hospital of the Sisters of Charity.*—We are gratified to announce that this admirably conducted charity has lately received from the state appropriation for hospitals out of the city of New York, nearly fourteen thousand dollars. This has enabled the trustees to purchase a large portion of the vacant lots surrounding the hospital, leaving a balance sufficient to erect another wing, which will considerably enlarge its accommodations for the sick. The new addition is under contract, and already in progress. The increase of room will render it practicable to isolate fever cases more effectually than has hitherto been possible owing to the limited number of wards. The flourishing condition of the institution is a source of great satisfaction to its numerous friends.

